# Severity and progression rate of periodontitis are associated with an increased risk of hypertension of patients attending a university clinic

**DOI:** 10.1186/s12903-022-02637-w

**Published:** 2022-12-22

**Authors:** Burak G. Yildirim, Cemilenur Aksit, Mesut Mutlu, Mari Ainola, Kari K. Eklund, Jaakko Leskelä, Pirkko Pussinen, Arzu Beklen

**Affiliations:** 1 Department of Periodontology, Faculty of Dentistry, Eskisehir Osmangazi University, Eskisehir, Turkey; 2 Faculty of Dentistry, Erciyes University, Kayseri, Turkey; 3 Faculty of Dentistry, Ege University, Izmir, Turkey; 4 Department of Medicine, University of Helsinki and Helsinki University Hospital, Helsinki, Finland; 5 Translational Immunology Research Program (TRIMM), Research Program Unit (RPU), University of Helsinki, Helsinki, Finland; 6 Inflammation Center, Division of Rheumatology, Helsinki University Hospital, Helsinki, Finland; 7 ORTON Orthopaedic Hospital of the Orton Foundation, Helsinki, Finland; 8 Oral and Maxillofacial Diseases, University of Helsinki, Helsinki, Finland; 9 Institute of Dentistry, University of Eastern Finland, Kuopio, Finland

**Keywords:** Hypertension, Blood pressure, Oral health, Periodontitis, Classification, Public health

## Abstract

**Background:**

Although periodontitis is associated with increased risk of hypertension, studies based on new periodontal disease classification is limited. We investigated whether periodontitis severity and progression rate are linked with self-reports on doctor-diagnosed hypertension in a large cohort of patients attending the periodontology clinic at the faculty of dentistry.

**Methods:**

Archived patient files, including radiographic image records and results from full-mouth clinical periodontal examination were screened for inclusion. Data on socioeconomic factors, smoking and oral hygiene habits, and medical history were collected with a questionnaire.

**Results:**

Diagnosis and background data were available for 7008 patients. The median (IQR) age was 31.0 (21.0) years; 60.1% (*n* = 4211) were female. Hypertension was diagnosed in 6.2% (*n* = 435) of patients. Both periodontitis stage and grade differed (*p* < 0.001) between patients with or without hypertension. Increased periodontal disease severity was associated with a 20% increasing risk for hypertension; the odds ratio (OR) was 2.63 (95% confidence interval [CI] 1.48–4.68, *p* < 0.001) in stage IV periodontitis. Increasing periodontitis progression rate was associated with a 35% increased risk for hypertension; the OR was 2.22 (95% CI 1.45–3.40, *p* < 0.001) in grade C periodontitis.

**Conclusion:**

Severity and progression rate of periodontitis may be independent risk factors for hypertension in this large cohort of patients attending the university periodontal department.

## Background

Hypertension, also known as persistent high or raised blood pressure, is a common condition. Long-term increased force of blood against the artery walls may eventually lead to an increased risk of other systemic diseases, such as asymptomatic organ damage, cardiovascular disease, and chronic kidney disease [1]. According to the World Health Organization (WHO), hypertension is considered as the cause of 12.8% of total deaths worldwide [2].

In recent years, many clinical and observational studies have supported an immunologic basis behind hypertension. In this concept, high blood pressure promotes immune-cell activation and increases inflammatory mediators to promote tissue entry of activated inflammatory cells [3]. Thus, accumulated activated immune cells in circulation and tissues promote an inflammatory response that disrupts functions that regulate blood pressure, which in turn leads to hypertension. Indeed, chronic inflammatory disorders may provide a foundation for pro-hypertensive inflammation [3, 4]. Periodontitis is one of the most common inflammatory diseases of infectious origin that often evolves into a chronic condition. Aside from irreversible loss of periodontal tissues, periodontitis is an unresolved hyper-inflammatory condition that can cause impaired immune system function, dysbiosis of host microbiota and other problems associated with systemic health [5].

The relationships between the infectious, immune, inflammatory and systemic features of periodontitis and its many related diseases are poorly understood. Despite contradictory results and explanations, most studies have revealed a significant association between periodontal diseases and hypertension [5, 6]. Regardless of the gaps in knowledge [7], a recent comprehensive review concluded that improved oral hygiene and periodontal therapy may prevent arterial hypertension and potentiate its treatment [8]. Periodontitis is associated with a 22–49% increased risk of arterial hypertension and may involve an approximately 20% higher risk of ineffective and unsuccessful treatment [9]. Furthermore, the risk for hypertension is increased by 16–67% by severity of periodontitis from moderate to severe [6, 8–10].

Earlier studies are based on a periodontal disease classification system including slight, moderate, and severe periodontitis [11]. The classification of periodontal diseases was changed in 2017 [12]. The new periodontal classification presents a staging and a grading, which classifies the severity and extent of the condition based on the measurable amount of destroyed or damaged tissue (or both) and the rate of disease progression. The main aim of this study was to analyze the relationship between periodontitis using the new classification with staging and grading and hypertension. In this study, the periodontitis diagnosis was based on the classification scheme from the 2017 World Workshop on the Classification of Periodontal and Peri-Implant Diseases and Conditions.

## Methods

### Subject and data collection

We investigated a large cohort of patients who were referred to the clinic of the Department of Periodontology in Turkey. This cross-sectional study examined the records of patients (*n* = 7008) diagnosed with a periodontal disease in 2015–2019. They were patients of the Eskisehir Osmangazi University, Faculty of Dentistry, who were referred to the clinic of the Department of Periodontology. Dental trainees performed complete periodontal examinations on each individual under the supervision of four specialized dentists. Each dental student received a clinical training on procedure before examining the patients. Style of collecting data and probing was determined by department agreement for each individual probing site. The Williams periodontal probe (Hu-Friedy, Chicago, IL USA) was used for periodontal probing. Archived patient files and radiographic image records were screened for inclusion in this analysis. The Ethics Committee of Eskisehir Osmangazi University approved the study (Ethical permit: 2021–52).

The archived data consisted of patient’s sociodemographic factors (sex, age, occupation andeducation), periodontal parameters, medical history, oral hygiene habits, smoking, reasons for appointment and digital radiographs. Periodontal disease groups were recorded based on the official manuscript from the 2017 World Workshop on the Classification of Periodontal and Peri-Implant Diseases and Conditions presented by the American Academy of Periodontology (AAP) and the European Federation of Periodontology (EFP) [12]. The diagnosis was concluded based on criteria of severity and complexity for the stage, whereas grade was done according to the ratio of bone loss to age and risk factors. Periodontal diagnoses that were based on the former classification were re-evaluated and classified according to the new classification.

**Fig. 1:**
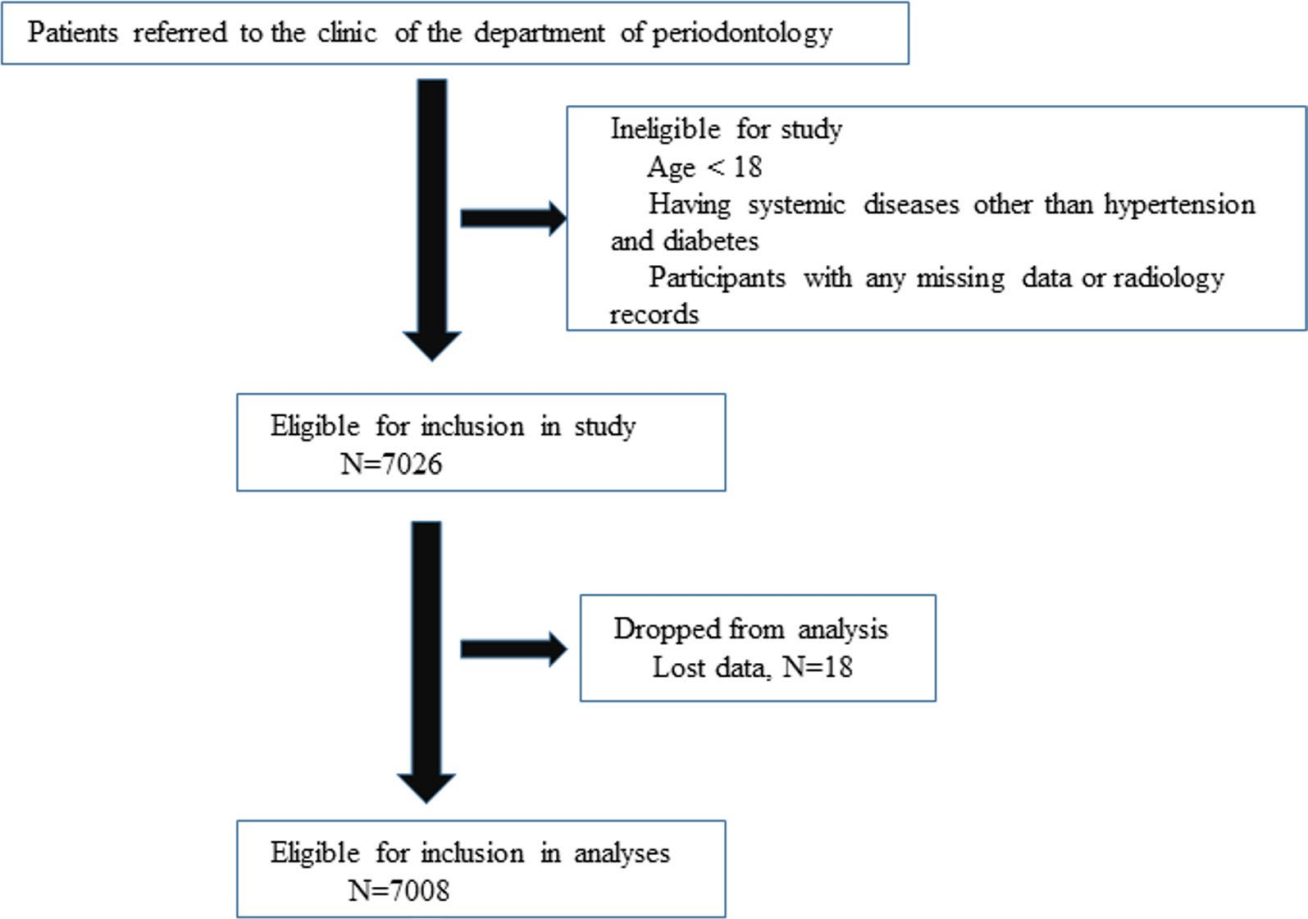
Flow chart. Showing the inclusion and exclusion criteria

Inclusion criteria for this study were age ≥ 18 years and having information on systemic medical history (diagnosis of hypertension, diabetes or both). Exclusion criteria were the presence of a systemic medical history other than hypertension or diabetes and absence of any required data or radiology records (Fig. 1).

### Periodontal parameters and diagnosis

Full-mouth periodontal records of each patient were obtained to determine periodontal health status. These records included the plaque index (PI) [13], gingival index (GI) [14], probing depth (PD) [15], clinical attachment level (CAL) and bleeding on probing (BOP). PI and GI were recorded at four sites per tooth, while PD and CAL were measured at six sites per tooth using a manual periodontal probe. For PD and CAL measurements, the highest values were considered for recording. BOP was considered positive if bleeding occurred within 30 s after probing. Alveolar bone loss was identified from periapical and panoramic radiographs radiographs of the entire dentition. We assessed data to determine if a missing tooth was extracted due to periodontal disease, tooth caries or trauma. Wisdom teeth were included in the count for missing teeth. Smoking, diabetes, and age recorded as risk factors for periodontal diseases.

Diagnosis of dental plaque-induced gingivitis (GIP) was based on the presence of gingival inflammation findings (such as BOP, oedema, hyperaemia) and pocket depth ≤ 3 mm but without radiographic bone loss and CAL. In contrast, patients with similar findings of GIP plus CAL were diagnosed as having plaque-induced gingivitis on a reduced periodontium (GRP).

According to the new disease classification the severity and extent of periodontitis was divided into four stages (I to IV). Briefly, we defined stage I as ≥ 2 interproximal sites with CAL = 1–2 mm (not on the same tooth) and radiographic bone loss at coronal third (< 15%) and stage II as ≥ 2 interproximal sites with CAL = 3–4 mm (not on the same tooth) and radiographic bone loss at coronal third (15–30%). Neither stage included tooth loss due to periodontitis. In stage III and stage IV, the patients had the interdental CAL ≥ 5 mm and radiographic bone loss extended to the middle third of the root and beyond. The difference between stage III and IV was based on the number of lost teeth (stage 3, ≤ 4lost teeth; stage 4, ≤ 5 lost teeth). We next assessed the rate of progression (Grade A, B and C) by measuring the radiographic bone loss in percentage of root length divided by the age of the subject (% bone loss-/-age) [16].

### Measurement of variables

In addition to gender, sex and age, the archived questionnaire included occupation and education status. Age was stratified into five groups (18–25, 26–34, 36–45, 46–55 and ≥ 56 years). Diagnosis of hypertension was assessed via questionnaire as follows: “Have you been diagnosed by your doctor for any of the following (*check all that apply*) **□**Hypertension/High blood pressure, □Diabetes mellitus”. Only patients who were diagnosed or treated by a physician were accepted for the study. Additional study variables collected via the same questionnaire included the patient’s oral hygiene practices, smoking habits and reasons for appointment. Smoking status was determined with the standard National Health Interview Survey of the U.S. Public Health Service (NHIS) [17] current smoking definition, which screens for lifetime smoking ≥ 100 cigarettes were used for grouping (never, current, former, environmental smoker). Oral hygiene practices were also determined based on the frequency of daily teeth brushing and flossing.

### Statistical analysis

Analyses were performed with IBM SPSS (version 28). Categorical variables are presented as numbers and percentages. Statistical significance of the differences was analysed by χ2 test. Multivariate logistic regression analysis was used to test the association between hypertension and periodontal disease diagnoses. After crude models, age and sex, presence of diabetes, smoking status and level of education (to describe socioeconomic status) were used to adjust the models. As smoking and diabetes are considered when defining grades of periodontitis (A, B and C), these variables were not used in the logistic regression to adjust the models. A *p*-value < 0.05 was considered statistically significant.

## Results

We evaluated the periodontal status of all available patients attending the periodontal clinic of the University of Eskisehir Osmangazi, Turkey, during years 2015–2019. Altogether, there were 7008 patients. The main original reasons for the appointment at the Institute of Dentistry were clinical oral examinations (19.0%), scaling or periodontal control (29.1%), endodontic pain or dental abscesses (10.8%) and caries (10.1%) (Table 1). Most patients were between 18 and 45 years of age (*n* = 5476, 78.1%); median (IQR) age of the whole cohort was 31.0 (21.0) years and females (*n* = 4211, 60.1%). Patients (*n* = 5398; 77.0%) reported brushing once or twice a day, only 16.4% (*n* = 1152) reported flossing.

**Table 1:** Characteristics of the cohort attending the periodontal clinic

Characteristic			All, *n* = 7008
			*n* (%)
Age groups (years)	18–25		2552 (36.4)
	26–35		1522 (21.7)
	36–45		1402 (20.0)
	46–55		943 (13.5)
	> 56-		589 (8.4)
Gender	Females		4211 (60.1)
Main indications for appointment	Check-up		1334 (19.0)
	Scaling or periodontal control		2040 (29.1)
	Endodontic pain or dental abscess		758 (10.8)
	Caries or filling		708 (10.1)
	Gingival bleeding		476 (6.8)
	Impacted third molars		452 (6.4)
	Prosthetics		397 (5.7)
Oral hygiene habits	Flossing		1152 (16.4)
	Brushing	Cannot brush	170 (2.4)
		Less than once a day	991 (14.1)
		Once a day	2112 (30.1)
		2 times a day	3286 (46.9)
		3 times a day	449 (6.4)

**Fig. 2:**
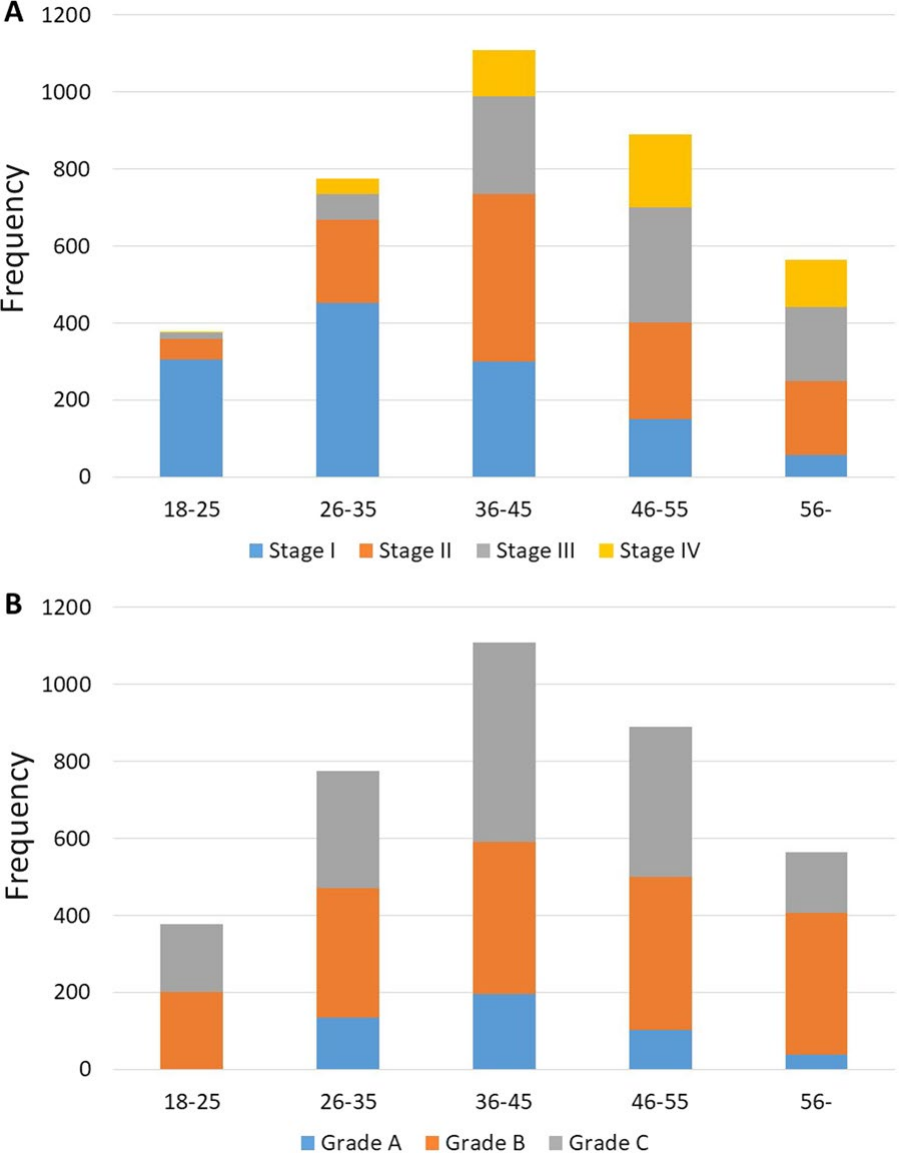
Periodontal disease diagnosis according to the age groups. Among the 7008 patients attending the department of periodontal diseases, 3718 (53.1%) were diagnosed with periodontitis. The figure shows the numbers in the age groups with different **A** stage and **B** grade of periodontitis

A total of 3290 (46.9%) patients had gingivitis; of these, 2506 (76.2%) had diagnosis on intact periodontium and 784 (23.8%) on reduced periodontium. Thus, 3718 (53.1%) patients were diagnosed with periodontitis. Figure 2 presents stage and grade according to age groups (Fig. 2). Stage I, stage II, stage III and stage IV were observed in 1264 (34.0%), 1146 (30.8%), 830 (22.3%), and 478 (12.9%) patients, respectively. Stage I was most common in the age group 26–35 years (*n* = 452, 35.8%); stage IV was most common in the age group 46–55 years (*n* = 188, 39.3%) (Fig. 2A). Grade A, grade B and grade C were observed at frequencies of 12.7% (*n* = 471), 45.6% (*n* = 1695) and 41.7% (*n* = 1552), respectively (Fig. 2B). Grade A was most frequent in the age group 36–45 years (*n* = 196, 41.6%), grade B in the age group 46–55 years (*n* = 398, 23.5%) and grade C in the age group 36–45 years (*n* = 520, 33.5%).

**Fig. 3:**
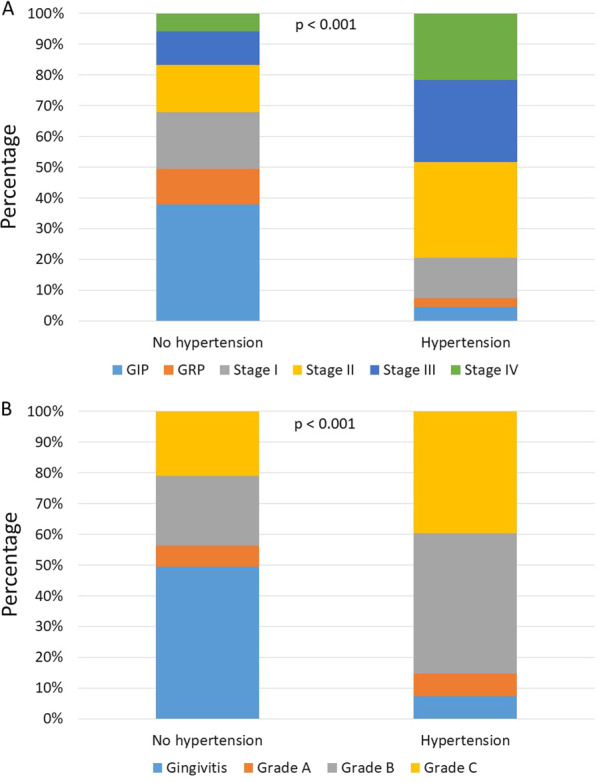
Periodontal disease diagnosis according to the presence of hypertension. Among the 7008 patients, attending the department of periodontal diseases, 435 (6.2%) had been diagnosed with hypertension. Panel **A** shows the periodontal disease diagnosis of patients without and with hypertension. Panel **B** shows the grade of periodontitis in patients without and with hypertension. The *p*-values depict the statistical significance produced by χ^2^ test

Four hundred thirty-five (6.2%) patients had doctor-diagnosed hypertension. The distribution of periodontal disease diagnoses differed (*p* < 0.001) between patients with or without hypertension (Fig. 3A). The most frequent diagnosis was GIP (*n* = 2486, 37.8%) among patients without hypertension. Patients with hypertension most frequently had stage II periodontitis (*n* = 136, 31.3%). The least common diagnosis among patients without hypertension was stage IV periodontitis (*n* = 384, 5.8%). The least common diagnosis was GRP (*n* = 12, 2.8%) among patients with hypertension. In addition, periodontitis grades differed between patients with or without hypertension (*p* < 0.001) (Fig. 3B). The most common periodontitis grade in patients with hypertension (*n* = 199, 49.4%) or without (*n* = 1496, 45.1%) hypertension was B.

The available risk factors for hypertension are presented in Table 2. As expected, the age distribution of patients without or with hypertension differed significantly (*p* < 0.001); the median (IQR) ages were 30.0 (20.0) and 55.0 (14.0) years, respectively. Patients with hypertension more frequently (*p* < 0.001) had diabetes, whereas patients without hypertension were more often smoking (*p* < 0.001). The proportion of ever smokers was 24.6% or 34.2% among patients with or without hypertension respectively. Patients with hypertension also more often had only basic education or were unemployed.

**Table 2:** Available hypertension risk factors of patients with and without hypertension

Risk factor		No hypertension *n* = 6573	Hypertension *n* = 435	*p*-value^1^
		N (%)		
*Age (years)*				
	18–25	2542 (38.7)	10 (2.3)	** < 0.001**
	26–35	1512 (23.0)	10 (2.3)	
	36–45	1345 (20.5)	57 (13.1)	
	46–55	791 (12.0)	152 (34.9)	
	> 56-	383 (5.8)	206 (47.4)	
*Sex*	Female	3935 (59.9)	276 (63.4)	0.140
*Smoking*	Current smoker	2014 (30.6)	79 (18.2)	** < 0.001**
	Ex-smoker	68 (1.0)	6 (1.4)	
	Passive smoker	168 (2.6)	22 (5.1)	
	No	4323 (65.8)	328 (75.4)	
*Diabetes*		196 (3.0)	116 (26.7)	** < 0.001**
*Education level*				
	Primary and middle school	1097 (16.7)	209 (48.0)	** < 0.001**
	High school	1968 (29.9)	135 (31.0)	
	Vocational education or university	3314 (50.4)	81 (18.6)	
	Post-graduate	194 (3.0)	10 (2.3)	
*Working status*				
	Student	2060 (31.3)	10 (2.3)	** < 0.001**
	Employee	2513 (38.2)	109 (25.1)	
	Unemployed	1655 (25.2)	177 (40.7)	
	Retired	339 (5.2)	137 (31.5)	

^1^ χ^2^ test; statistically significant differences are in bold

The association between hypertension and periodontal disease diagnosis was analysed with logistic regression models, including crude model, age- and gender-adjusted model, and fully adjusted model (Table 3). Compared to the diagnosis GIP, the patients with stage II, stage III, or stage IV periodontitis had an independent odds ratio (OR) of 1.92 (95% confidence interval [CI] 1.13–3.29; *p* = 0.017), 1.78 (95% CI 1.03–3.09; *p* = 0.039) and 2.63 (95% CI 1.48–4.68; < 0.001) for hypertension, respectively. Changing from a diagnosis group to another with increasing severity was associated with an OR of 1.20 (95% CI 1.09–1.32; *p* < 0.001) for hypertension. In a fully adjusted model, grade C periodontitis was associated with hypertension (OR 2.22, 95% CI 1.45–3.40; *p* = 0.001). Increased disease progression from grade to another was associated with an OR of 1.35 (95% CI 1.19–1.53; *p* < 0.001) for hypertension.

**Table 3:** Association between periodontitis and hypertension

		OR (95% CI), *p* value		
Diagnosis of periodontal disease		Model 1	Model 2	Model 3
Gingivitis	GIP	1.0	1.0	1.0
	GRP	1.93 (0.94–3.97), 0.073	1.14 (0.54–2.42), 0.739	0.95 (0.43–2.10), 0.904
Periodontitis	Stage I	**5.85 (3.51–9.81), < 0.001**	1.58 (0.91–2.73), 0.105	1.42 (0.81–2.49), 0.222
	Stage II	**16.7 (10.4–26.9), < 0.001**	**1.97 (1.16–3.32), 0.012**	**1.92 (1.13–3.29), 0.017**
	Stage III	**20.2 (12.5–33.0), < 0.001**	**1.77 (1.03–3.02), 0.037**	**1.78 (1.03–3.09), 0.039**
	Stage IV	**30.4 (18.6–50.0), < 0.001**	**2.68 (1.54–4.65), < 0.001**	**2.63 (1.48–4.68), < 0.001**

Diagnosis of periodontal disease		Model 4	Model 5	Model 6
Gingivitis		1.0	1.0	1.0
Periodontitis	Grade A	**7.42 (4.50–12.2), < 0.001**	1.24 (0.72–2.14), 0.437	1.21 (0.70–2.10), 0.488
	Grade B	**13.5 (9.28–19.8), < 0.001**	1.41 (0.91–2.19), 0.121	1.36 (0.88–2.11), 0.169
	Grade C	**12.7 (8.66–18.6), < 0.001**	**2.34 (1.54–3.56), < 0.001**	**2.22 (1.45–3.40), < 0.001**

Logistic regression models hypertension (yes/no) as dependent

Statistically significant associations are in bold

Model 1, crude; Model 2, age (years), gender; Model 3, age (years), gender, education level, smoking (never/ever), DM (no/yes)

Model 4, crude; Model 5, age (years), gender; Model 6, age (years), gender, education level. GIP: dental plaque-induced gingivitis, GRP:plaque-induced gingivitis on a reduced periodontium

## Discussion

In this large cohort of patients attending the university periodontal department, periodontitis stage and grade were independent risk factors for hypertension. Increased periodontal disease severity was associated with a 20% increased risk for hypertension; the risk increased 2.6-fold in stage IV periodontitis. Increasing periodontitis progression rate was associated with a 35% increased risk for hypertension; the risk increased 2.2-fold in the most progressive form of periodontitis.

Several studies have suggested an association between periodontal diseases and hypertension, as summarized in systematic reviews [6, 9]. A quantitative meta-analysis comprising 40 cross-sectional studies revealed that moderate-severe and severe periodontitis were associated with hypertension (OR 1.22, 95% CI 1.10–1.35 and OR 1.49%95 CI 1.09–2.05, respectively [9]. Although the summary estimates included both confident and non-confident periodontitis diagnosis, including only confident diagnosis did not considerably increase the risk. Thus, these estimates are comparable but somewhat lower than those found in the present study, which is based on the new classification of periodontitis severity.

One study using Mendelian randomization suggested a causal relationship between periodontitis and hypertension, as genetic variants associating with periodontitis recognized in GWAS were also linked with blood pressure phenotypes [4]. In two longitudinal studies, childhood gingival inflammation was present at the same time as increased diastolic blood pressure and preceded increased systolic blood pressure in adulthood [18, 19]. The evidence suggesting that periodontal therapy reduces blood pressure is inconclusive [9, 20] due to the scarcity of relevant studies [4]. Hypertension is independently associated with multi-morbidity [21]. Furthermore, hypertensive patients with periodontitis have an increased risk of several systemic diseases, especially CVD and respiratory disease [22]. The recognized association between periodontitis and hypertension, and the possibilities for interventions are essential considering the high prevalence of both diseases and increased societal burden of underdiagnosed and undertreated periodontitis.

The main limitations of this study include missing information on the main risk factors for hypertension [23]. These comprise modifiable risk factors, such as diet (excessive salt or saturated fat intake or alcohol consumption), physical inactivity and obesity. For non-modifiable risk factors, we did not have information on family history of hypertension. On the other hand, we considered age, sex, diabetes, smoking habits and socioeconomic status. In addition, the age distribution of the patients was highly skewed, including a high proportion of young individuals, whereas hypertension mainly affected patients ≥ 46 years of age (Table 2). Since all participants were patients of the periodontal department, our cohort did not include any participants with entirely healthy periodontium and the reference group consisted of patients with mild gingivitis on intact periodontium. We did not have information on blood pressure levels, since the hypertension diagnosis was based on self-reporting on a doctor-diagnosed condition. Validity of self-reports on chronic conditions may vary between countries and settings and is a source of bias. In 2012, 55% of Turkish hypertensive patients were aware of their diagnosis and the treatment rates have increased since 2003 [24]. Thus, in the present study the proportion of patients with hypertension may be underestimated. The cohort may include “false negatives” in terms of hypertension, but presence of “false positive” patients is unlikely. Furthermore, other factors, such as systemic medications may contribute to periodontal diseases. However, this study only assessed the presence of hypertension without recording the data on medication, thus the association can not be ruled out from the influence of medication side effects. Besides that, the current literature remains inconclusive regarding the effect of medications, as with no or positive associations are reported conclusively [25]. Such as, thiazide-like diuretic, which is one of the first line antihypertensive therapies, contributes to the development of periodontal disease [26] by reduced salivary rate and composition [27]. On the contrary, use of angiotensin-converting enzyme inhibitors does not show an association with periodontitis [25]. The main strength of our study is the large cohort size with a careful periodontal disease diagnosis including the severity and the estimated progression rate [28].

In addition to shared genetic susceptibility mechanisms combining periodontitis and hypertension may derive from dysbiosis, which is characterized as an imbalance of microbiota that changes its functional composition and metabolic activities and thus plays an important role in health and disease [29]. Despite varying compositions in different body sites, it is plausible that microbiotas are interconnected not only with host cells but also with each other [30]. In addition, due to the complex interplay between the microbiome and the host, host genetics and genetic interactions with environmental factors (such as diet) presumably affect microbiota composition [31]. Changes in gut microbiota may lead to increased gut permeability (so-called leaky gut) and dissemination of microbes and microbe-derived molecules, such as endotoxins [32]. The subsequent pathological responses by the host result in various metabolic diseases, including hypertension [33]. In addition to inflammation, dysbiosis is the main feature of periodontitis [34]. Oral bacteria may translocate easily via bleeding gums, lymph, immune cells, or saliva [32]. Interestingly, oral bacteria may change the gut microbiome, modify immune defence, and increase gut permeability [35–37]. Thus, future studies may determine if oral and gut microbiota are interconnected and whether dysbiosis is a driver of cardiometabolic diseases.

## Conclusions

WE show how severity and potential progression rate of periodontitis may be associated with hypertension. An increased risk of hypertension was observed already in mild periodontitis and the risk increased significantly with increasing severity. However, gingivitis was not associated with risk for hypertension. Periodontitis is a gradual process that takes place over many years and always starts with gingivitis. Proper treatment of gingivitis usually reverses symptoms and prevents its progression to periodontitis, which is associated with higher hypertension rate. Thus, early prevention and oral health care services should be implemented in health policies to decrease the burden of chronic diseases, such as hypertension.

## Data Availability

The datasets used and/or analysed during the current study available from the corresponding author on reasonable request.

## Abbreviations

AAP: American academy of periodontology
BOP: Bleeding of probing
CAL: Clinical attachment level
CI: Confidence interval
EFP: The European federation of periodontology
GI: Gingival index
GIP: Dental plaque-induced gingivitis
GRP: Plaque-induced gingivitis on a reduced periodontium
PD: Probing depth
PI: Plaque index
NHIS: U.S. Public Health Service
